# The lived experiences of patients with cancer during the COVID-19 pandemic: a qualitative study

**DOI:** 10.3332/ecancer.2023.1598

**Published:** 2023-09-04

**Authors:** Gracia Fahed, Angie H Fares, Aya Ghosn, Alain Greige, Elsa Hebbo, Kim Naja, Pamela Moukarzel, Salame Haddad, Antoine Finianos, Gladys Honein, Elie A Akl

**Affiliations:** 1 American University of Beirut Faculty of Medicine, Beirut 1107, Lebanon; 2 American University of Beirut Hariri School of Nursing, Beirut 1107, Lebanon; † Equal contribution

**Keywords:** cancer, COVID-19, lived experience

## Abstract

**Background:**

The objective of this study was to explore the impact of COVID-19 on the lived experiences of patients with cancer in Lebanon.

**Methods:**

We adopted a descriptive phenomenological approach. We included adults who had been diagnosed with cancer before the pandemic and undergoing treatment at the American University of Beirut Medical Centre. We conducted virtual, semi-structured in-depth interviews with either video or audio recordings. Two team members coded the transcripts independently and identified common themes and patterns.

**Results:**

We recruited 11 participants for the study. The analysis identified the following six themes: perceived seriousness of COVID-19, fear of COVID-19 versus fear of cancer, coping mechanisms, treatment availability and accessibility, compliance with public health and social measures and precautionary measures in the healthcare system. The coping mechanisms included staying positive, seeking normalcy, using family support, religiosity and fatalism.

**Conclusion:**

Faced with many challenges during the COVID-19 pandemic, patients with cancer resorted to a range of coping strategies.

## Introduction

Two years and 5 months following the emergence of the first cases of severe acute respiratory syndrome coronavirus 2 in Wuhan, China [16], the world is still suffering from the repercussions of what was declared a global pandemic on 11 March 2020 [5]. National lockdowns were imposed [28], work-from-home increased heavily [3], various technologies were strategically employed to minimize the hazards and repercussions of the disease [13], and relentless efforts to develop treatments were deployed [9]. In the background, the reluctance of people to receive recommended immunisation was already growing even before the vaccines became available [19].

A large number of studies have investigated the lived experiences of people during the pandemic. Most of these studies explored the experiences of frontliners who are directly exposed to the virus. Recurrent findings included psychological distress, depression, anxiety [6, 24], fear of death [15] and frustration with public ignorance of preventive measures [12]. Nurses and physicians reported exhaustion due to heavy workload, fear of becoming infected and infecting others and feeling powerless to handle patients’ conditions [17]. Concomitant positive emotions included growth under pressure, development of professional responsibility and self-reflection [27]. Negative feelings related to isolation were a major culprit affecting the coping capacity and self-esteem of COVID-19 patients who are not members of the healthcare system [23].

Patients with cancer have faced multiple obstacles that made them particularly vulnerable during the pandemic. They have suffered from treatment delays (surgery, chemotherapy, radiotherapy, etc.) that negatively affected their outcomes. A study conducted in the United Kingdom found that a delay in cancer surgeries for 3 months is predicted to account for almost 5,000 excess deaths [18]. Patients with cancer have refrained from visiting their physicians out of fear of contracting the infection [30]. They also feared the infection would have more severe consequences for them compared to healthy individuals [29]. In a survey study, patients with sarcoma reported cancer-related worry, low-resilience coping and uncertainty about treatment intent in relation to the COVID-19 pandemic [31]. Patients with terminal cancer may also be concerned about complicated grief reactions of their families unable to say ‘goodbye’ because of ‘no visitor’ institutional policies [30].

Lebanon is a developing country where the COVID-19 pandemic was compounded with severe political, economic and financial crises, social unrest, and what came to be known as the ‘Beirut blast’, the largest non-nuclear explosion in modern history [8]. The objective of this study was to explore the impact of COVID-19 on the lived experiences of patients with cancer in Lebanon.

## Methods

### Study design

We adopted a descriptive phenomenological approach. Descriptive phenomenology is a qualitative research strategy that focuses on individuals’ thoughts, perceptions and emotions regarding a specific event [22]. The in-depth interviews allow the researcher to get a deeper understanding and appreciation of the experience of the participant [22].

### Study participants

We included adults who had been diagnosed with cancer before the pandemic and undergoing treatment at the American University of Beirut Medical Centre (AUBMC). They could have experienced or not COVID-19 illness. We excluded patients in palliative care.

### Sampling and recruitment

We used purposeful sampling to recruit the participants [21], whereby a treating physician informed potentially eligible patients about the study. The research team members then contacted interested patients and asked for verbal consent. The recruitment process occurred between 1 March and 30 March 2021. The study was approved by the American University of Beirut Institutional Review Board.

### Data collection

The team members conducted virtual, semi-structured in-depth interviews with either video or audio recordings. The interview guide included open-ended questions addressing: patients’ perception of the COVID-19 pandemic; the care they received during the pandemic; and their overall experience during this period, including worries, challenges and coping mechanisms among others. The interviews were conducted in Arabic.

### Data analysis

We analysed the data based on Giorgi’s method used in phenomenology [14]. We transcribed verbatim the audiotaped interviews in Arabic and then translated them into English. After carefully reading the transcripts several times, we selected and coded all the statements that are relevant to the phenomenon we are studying and categorised them into common themes across the texts. We then deciphered the findings generated in terms of similarities and differences in the participants’ experiences and identified common patterns. Two team members analysed the transcripts independently, then they reviewed their work together to ensure the coding and themes are consistent.

## Results

**Figure 1.**
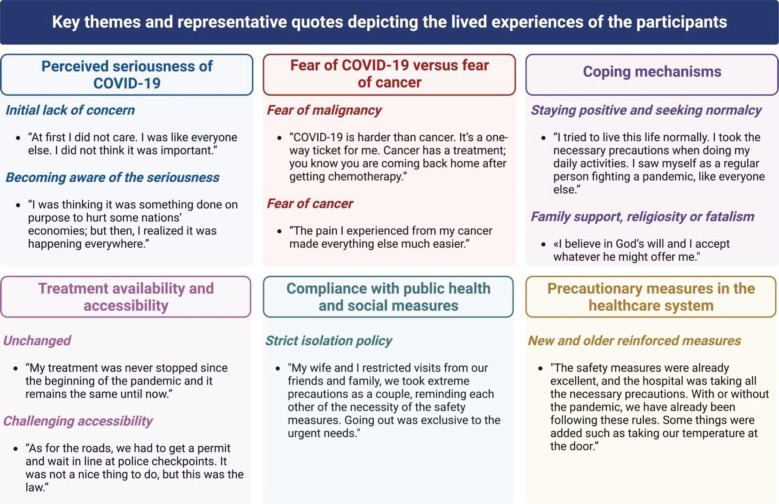
Key themes and representative quotes depicting the lived experiences of the participants.

We recruited 11 participants for the study (Table 1 for their demographic characteristics). The interviews lasted between 17 and 52 minutes. The analysis identified the following six themes that we describe in the subsequent sections: perceived seriousness of COVID-19, fear of COVID-19 versus fear of cancer, coping mechanisms, fatalism, treatment availability and accessibility, compliance with public health and social measures and precautionary measures in the health care system (Figure 1, thematic schema). Figure 1 presents the identified themes with representative quotes on the impact of COVID-19 on the lived experiences of patients with cancer in Lebanon.

### Perceived seriousness of COVID-19

The perception of COVID-19 evolved over time. Initially, most participants did not appreciate the seriousness of the detrimental effects of the virus on health. As time passed, participants were increasingly exposed to information from different sources (e.g., media, hospitals and professionals) leading to a better understanding of science but sometimes mixed with skepticism. Eventually, with more robust information and with close individuals experiencing the illness, most participants exhibited a greater level of trust in science and were relying on information from their healthcare providers and hospitals.

- ‘At first, I did not care. I was like everyone else. I did not think it was important’.
- ‘I was thinking it was something done on purpose to hurt some nations’ economies; but then, I realised it was happening everywhere’.
- ‘Unfortunately, for some people, COVID-19 is political, but this is not true as we are really seeing people getting infected and dying. My own daughter got infected’.

### Fear of COVID-19 versus fear of cancer

One participant was more worried about COVID-19 due to uncertainties about the disease and its treatment options. However, the majority feared cancer more than COVID-19, because COVID-19 was preventable, had less severe symptoms and had limited individual impact.

- ‘COVID-19 is harder than cancer. It’s a one-way ticket for me. Cancer has a treatment; you know you are coming back home after getting chemotherapy’.
- ‘I tried everything to treat my cancer, but I cannot control it. You have the option to control the effect of COVID-19 on you. You could take precautions that protect you. You do not have this with cancer’.
- ‘The pain I experienced from my cancer made everything else much easier’.

The fear reported by most participants stemmed from several causes: suffering and pain from COVID-19 symptoms, combating two diseases in the hospital, transmitting the virus to family and other cancer patients and exposing companions during chemotherapy treatment. The COVID-19-related fear increased over time for most participants due to increased community spread, accruing a number of deaths, coupled with low precautionary measures, more immunocompromised susceptibility and scarce robust evidence about COVID-19.

- ‘Every day they change the regulations about what should and should not be done to protect ourselves; it was so chaotic’.
- ‘I am afraid because of the side effects that are happening. The suffering the people are enduring is very terrifying’.
- ‘I think of the people who are taking chemotherapy with me. I do not want them to be in danger because I infected them’.
- ‘I was not afraid at first, but in the recent 2–3 months when the cases increased, I was surely afraid’.

One patient reported testing positive for COVID-19 3 days before her cancer surgery. She was worried that contracting the virus would delay her surgery and potentially lead to a worsening of her cancer. She was not anxious about the consequences of contracting COVID-19 but about finishing the 14 days of quarantine to be able to undergo the surgery.

- ‘My only fear during that time was that my cancer would metastasize’.

### Coping mechanisms

The participants reported different coping mechanisms to deal with the pandemic: (1) staying positive, (2) seeking normalcy, (3) using family support, (4) religiosity and (5) fatalism.

First, participants emphasised the vital importance of cultivating a positive outlook as a powerful coping mechanism for dealing with the challenges posed by both the pandemic and the diagnosis of cancer.

- ‘Dealing with either cancer or COVID-19 needs optimism and hope’.
- ‘I tried to deal with the pandemic with a cheerful mindset while trying to be cautious without being paranoid’.

Second, numerous participants described engaging in their daily activities as a way to regain a sense of normalcy:

- ‘I used to go to work and teach face-to-face classes until the very last day before lockdown. Even after that, I have never missed a single day of work online. This helped me feel stronger’.
- ‘I tried to live this life normally. I took the necessary precautions when doing my daily activities. I saw myself as a regular person fighting a pandemic, like everyone else’.
- ‘Daily, I walk outdoors while taking the necessary precautions’.
- ‘Sports are my daily way of venting, especially if the family’s children are home’.
- ‘I enjoy watching series and YouTube videos or doing some handcrafting activities whenever I feel good after treatment sessions’.
- ‘I search for activities that enhance my calmness and help me escape daily problems. Drawing, writing, solving puzzles, listening to music and gardening/planting are my go-to activities’.

Third, many participants used family support but feared the consequences on their health. Thus, many coped by connecting more with their close circle.

- ‘I felt the need of being surrounded, but I used to fear contracting the virus from visiting family members or neighbours’.
- ‘I spend my time at home with my daughters. We watch TV, I call my brothers’…
- ‘I constantly phone call my son who’s studying abroad, and it brings me grace and safety’.
- ‘My mother is my source of comfort and safety’.
- ‘Video calls are now a substitute for the family visits, which we do regularly’.

Fourth, the majority of participants reported that faith in God and prayer considerably helped them to cope with the pandemic. Their faith in the presence of a stronger power or saviour boosted their strength and provided them with a sense of inner peace.

- ‘My faith gave me the strength to cope with the situation’.
- ‘God saved me previously from my cancer diagnosis, so I have strong faith in him’.
- ‘Praying before each treatment session or test deeply alleviated my pain. I became even more faithful after the start of the pandemic’.
- ‘Prayer is my main coping mechanism. I regularly pray for the deceased and their families with whom I cannot be physically present to offer my condolences’.

Whether implicitly or explicitly stated, participants declared their acceptance of *God’s will.*

- ‘I believe in God’s will and I accept whatever he might offer me. This brings me some kind of inner peace to fight whatever situation’.
- ‘Whenever existential questions haunted me or whenever I had doubts regarding my diagnosis and the pandemic, my faith was the tool to find relief. I believe in God’s will and plan.
- ‘I do what I have to do, and the rest is on God’.

Fifth, many participants felt powerless facing the pandemic, which led to a sense of fatalism.

- ‘We don’t have a choice but to accept the current situation’.
- ‘I take all the necessary precautions and the rest is a matter of fate’.

‘If odds were for me to encounter the virus, then I cannot do anything about it’.

### Treatment availability and accessibility

For many patients, there was no change in treatment availability and accessibility in comparison to before the pandemic.

- ‘My treatment was never stopped since the beginning of the pandemic and it remains the same until now’.

For others, the pandemic negatively impacted treatment accessibility. Patients had to obtain a commuting permit for every trip during times of total lockdown.

- ‘As for the roads, we had to get a permit and wait in line at police checkpoints. It was not a nice thing to do, but this was the law’.

Some participants experienced difficulties with transportation to and from the hospital, adding sources of stress. In contrast, other participants found it easier to get to the hospital as there was no longer traffic on the roads.

### Compliance with public health and social measures

Some adopted a strict isolation policy whereby they imposed restrictions on visits from family members and friends.

- ‘My wife and I restricted visits from our friends and family, we took extreme precautions as a couple, reminding each other of the necessity of safety measures. Going out was exclusive to the urgent needs’.

The trust in science prompted one patient to adhere to all recommended precautionary measures and accept all potential health consequences.

### Precautionary measures in the health care system

Apart from the globally implemented public health and social measures, such as mandatory mask-wearing and practicing social distancing, the participants reported that the hospital applied additional supplementary measures during their visits. For example, the temperature of each patient was measured at the door. In addition, there was a private elevator for patients receiving chemotherapy to avoid exposure to other patients. The number of visitors was restricted for post-operative patients as well as during the chemotherapy sessions. Furthermore, the post-operative stay was shortened from 4 days before the pandemic to 1 day after it. Patients were also educated about the difference between COVID-19 symptoms and side effects of the chemotherapy which are strikingly similar. For many, measures such as wearing the mask and practicing social distancing remained largely consistent with pre-pandemic practices; they were just enhanced and reinforced. This was viewed as a source of comfort.

- ‘The safety measures were already excellent, and the hospital was taking all the necessary precautions. With or without the pandemic, we have already been following these rules. Some things were added such as taking our temperature at the door’.

The majority of participants perceived the precautions put in place by the hospital as something positive and comforting amidst a growing pandemic. In fact, the blind trust the patients put in the hospital helped alleviate their stress.

- ‘I was never afraid of going to the hospital. My mom would come with me and would always tell me to be careful and I would tell her not to worry and that they were already taking all the necessary precautions’.

## Discussion

### Summary of findings

Patients with cancer faced a number of challenges during the pandemic, including fear of COVID-19 and fear of cancer, treatment availability and accessibility, compliance with public health and social measures and precautionary measures in the healthcare system. To deal with these challenges, they used several coping mechanisms including staying positive, seeking normalcy, using family support, religiosity and fatalism.

### Strengths and limitations

Our study contributes to advancing the literature due to its many strengths. First, it is among the first studies that explore the lived experiences of cancer patients; a vulnerable population during a pandemic. Second, although a number of studies have highlighted the negative psychosocial impact of the pandemic on COVID-19 patients [10, 25], very few reveal the positive outcomes. In this study, a number of participants expressed their personal and social growth as they had a considerable amount of free time to reflect and appreciate life and its ephemerality, and more time to spend with family members who also became more involved with their health and more supportive in response to the outbreak. Third, this study highlighted the experience of cancer patients in the early phase of the pandemic, before vaccination campaigns started in Lebanon, and when a COVID-19 infection would be more infectious, more severe, and deadlier especially in cancer patients. This could serve as both a strength and limitation as their lived experience might be unique, capturing the initial months of social distancing and isolation, which would probably differ after worldwide vaccination and decreased COVID-19 hospitalisations.

Nevertheless, this study has some limitations that should be recognised. In addition to having a small-sized sample (11 cancer patients), the participants come from one sole healthcare centre (AUBMC) in one country in the middle east (Lebanon). This lack of diversity makes the study findings regionally limited and therefore the results should be carefully interpreted and generalised. Moreover, Lebanon is a developing country that suffered from severe financial and political crises amidst the COVID-19 pandemic, which would aggravate the fear and negativity felt by the participants as well as limit their capacity of experiencing some coping mechanisms.

### Comparison to other studies

Nawfal *et al* [7] conducted a survey study exploring the same question addressed in our research but focusing on other medical centres around Lebanon. Similarly, to our findings, the authors reported that the majority of patients expressed a greater fear of cancer than COVID-19, and encountered transportation challenges to and from the hospital. However, while in our study participants agreed that precautionary measures in both private and public hospitals were very well implemented, Nawfal *et al* [7] reported that approximately a quarter of participants experienced difficulty in maintaining precautionary measures in both private and public hospitals. This difference might be related to differing practices and standards between medical centres.

Regarding the coping mechanisms adopted by cancer patients during the pandemic, our study revealed that faith in God and prayer play a significant role for the majority of participants. These findings align with a previous study conducted by Ng *et al* [20] which demonstrated a link between anxiety and depression in cancer patients and negative religious coping, as well as lower non-organisational religiosity.

As a result, it becomes evident that interventions aimed at reducing religious struggles and promoting religious activities could greatly assist cancer patients in coping more effectively with their illness.

Overall findings were similar to what was found in a population of patients with end-stage renal disease on haemodialysis [26], namely with regard to the following narratives: impact of the pandemic on social relationships (isolation and decreased shared activities), fear of contracting COVID-19 due to being a high-risk population and increased anxiety and rumination. Also, similar to our study, positive outcomes were observed, namely the co-existence of distress and growth in the face of the pandemic. Patients were able to find meaning in their distress and developed coping strategies to manage it. These findings are consistent with Frankenhaeuser’s effort-distress model [11] which shows that high distress and high coping efforts may co-exist in the face of a perceived threat.

A study by Aydin *et al* [1] reporting the lived experiences of patients with gynaecological cancers during the pandemic showed similar findings to ours with regard to patients’ fear of cancer more than COVID-19 itself. These patients also coped with the combined stressors of cancer and COVID-19 by embracing religion [1]. In contrast to our patient population, however, patients with gynaecological cancers during the pandemic noted a reduction in the frequency of appointments to reduce the risk of COVID-19 transmission, as well as disruption in treatment services. Participants in our study had no barriers or delays in treatment and care.

Despite our study's participants not experiencing disruptions in their access to treatment and care during the pandemic, some of them expressed major concerns about the possibility of such issues arising. This concern is not unfounded, as research has shown that across eight hospitals in China, Italy and England, cardiovascular care witnessed a significant decline of 60%–100% during the COVID-19 pandemic. Consequently, many conditions went undiagnosed or untreated, leading to a substantial increase in avoidable mortality [2]. Additionally, data from the National Health Service (NHS) England revealed a significant backlog of planned surgeries due to COVID-19, with the number of patients waiting to receive treatment reaching 4.46 million in November 2020 [4]. Thus, ensuring convenient and organised access to care for individuals with health problems, not necessarily COVID-related, becomes crucial.

In Malaysia, Fnu *et al* [32] conducted a qualitative study that shed light on the considerable stress caused by the high costs associated with cancer treatments. As a coping mechanism, individuals sought financial assistance to alleviate the burden. While we did not specifically explore this aspect in our study, it is likely that financial challenges are more severe in Lebanon, especially considering the simultaneous occurrence of a severe economic and financial crisis during the pandemic. The widespread economic downturn has made it increasingly difficult for individuals to secure the necessary assistance for their cancer treatments. Hence, addressing the financial burden of cancer care in Lebanon becomes even more imperative in such challenging times.

### Implications for practice

Based on this study’s findings, several recommendations can be made to improve the emotional and relational unmet needs of cancer patients amidst the COVID-19 pandemic. Telehealth services should be provided to obtain accurate information and easy access updates about treatment processes during the pandemic, as this would alleviate the worry and fear reported by patients on COVID-19 and its impact on cancer course and management. In addition, the important role of psycho-oncology arises again in this study. Tele-psychological hotlines and peer support groups should be implemented to improve the psychosocial well-being of these patients.

### Implications for research

Future work can seek to portray the impact of the COVID-19 pandemic on a larger, more representative sample of different population groups. The literature would also benefit from studies elucidating the long-term coping and adaptations of cancer patients as the pandemic is still ongoing.

## Conclusion

This study was conducted in 2020 during the COVID-19 pandemic. Eleven adults with cancer were recruited and interviewed about their lived experiences during the pandemic. The study found that the perception of COVID-19 has been dynamic as most of the patients gained perspective over time which allowed them to overcome their skepticism about the disease. Yet, cancer remained the main source of fear for all participants, except one. For the only patient in the study who contracted COVID-19, her fear was not as much from the potential effects of the infection *per se*, as it were from the possible risk of metastasis should her surgery be postponed. Moreover, the participants resorted to various strategies to cope with their disease in the midst of the pandemic. The strategies seemed to differ with participants’ different personalities. Some found a positive mindset or pursuing their daily activities with a sense of normalcy to be helpful, while others tended to be more cautious, religious or fatalistic. Furthermore, the experience with the health care system differed from one participant to another. Some reported no change compared to before the pandemic, while others felt that the lockdown compromised their access to medical care. Some of the participants reported their appreciation of the precautionary measures taken in the hospitals as well as the educational material they received in order to distinguish between possible COVID-19 symptoms and the side effects of chemotherapy. They regarded these measures as a source of comfort.

## Conflicts of interest

None of the authors declare any conflicts of interest.

## Funding

This study was not funded.

## Figures and Tables

**Table 1.** Demographic and clinical characteristics.

Characteristics	Patients (*n* = 11)
Gender Male Female	5 6
Cancer type Colon Breast Lung Melanoma with metastasis to the lungs Breast with metastasis to the bone Metastasised ovarian cancer Colon cancer with metastasis to the uterus	2 1 2 1 3 1 1
Cancer status Remission Relapse Ongoing treatment	2 2 7
Plan of treatment Chemotherapy + oral drug as outpatient Surgery + chemotherapy Chemotherapy Surgery + chemotherapy + radiotherapy Unknown course of treatment	1 4 4 1 1
Frequency of healthcare system visit Once per week Once every 2 weeks Once every 3 weeks Unknown	2 3 4 2
Time of diagnosis (pre-COVID) 1 year ago 2 years ago 3 years ago 4 years ago 5 years ago 19 years ago Unknown	2 3 1 2 1 1 1
